# Thermal-Input-Induced Microstructural Evolution and Mechanical Response of Mg-Gd-Y-Zn-Zr Alloy Wires During Electropulsing Treatment

**DOI:** 10.3390/ma19143045

**Published:** 2026-07-15

**Authors:** Jinchao Zou, Yonglin Zheng, Miaomiao Zhang, Yu Liu, Shikai Xu, Shiwen Zhu, Xiangyu Gao, Zhiquan Huang

**Affiliations:** 1 School of Mechanical Engineering, Taiyuan University of Science and Technology, Taiyuan 030024, China; 2024004@tyust.edu.cn (J.Z.); zhengyl1999@163.com (Y.Z.); zhangmiaomiao1113@163.com (M.Z.); 19371664973@163.com (Y.L.); xushikai2026@163.com (S.X.); 18835345332@163.com (S.Z.); 2021103@tyust.edu.cn (X.G.); 2 Shanxi Provincial Key Laboratory of Intelligent Metallurgical Equipment and Rolling Technology, Taiyuan University of Science and Technology, Taiyuan 030024, China

**Keywords:** rare-earth magnesium alloy, electropulsing treatment, thermal input, microstructural evolution

## Abstract

**Highlights:**

- Current density controls thermal input during electropulsing treatment.
- The surface peak temperature increased from 207 to 497 °C.
- The 15 A/mm^2^ sample achieved 312.2 MPa UTS and 13.6% elongation.
- Moderate thermal input promoted LPSO fragmentation and homogenization.
- Excessive thermal input caused W-phase precipitation and grain coarsening.

**Abstract:**

To reveal the influence of pulsed current density on the microstructural evolution and mechanical properties of Mg-Gd-Y-Zn-Zr rare-earth magnesium alloy wires, extruded Mg-10Gd-3.4Y-1.3Zn-0.4Zr alloy wire was selected as the research material. By regulating the current density in the range of 12–20 A/mm^2^, the effects on temperature rise behavior, microstructural evolution, and mechanical properties were systematically investigated. The results show that as the current density increases from 12 A/mm^2^ to 20 A/mm^2^, the measured surface peak temperature rises from 207 °C to 497 °C, and the mechanical properties among the electropulsing-treated samples exhibit a trend of first increasing and then decreasing. Among these treated samples, the optimal combination of strength and ductility is achieved at a current density of 15 A/mm^2^, at which the tensile strength and elongation reach 312.2 MPa and 13.6%, respectively. Microstructural analysis indicates that appropriate pulsed electrical parameters promote the dissolution, fragmentation, and homogenized dispersion of block-shaped long-period stacking ordered (LPSO) phases, thereby optimizing the internal strain state and facilitating the activation of non-basal <c+a> slip. However, when the current density increases to 20 A/mm^2^, excessive thermal input leads to grain coarsening and a network-like W-phase precipitation, indicating that excessive energy input can lead to microstructural instability and mechanical degradation.

## 1. Introduction

Magnesium alloys have broad application prospects in aerospace, rail transportation, electronic communication, and lightweight structural components owing to their low density, high specific strength, excellent damping capacity, and electromagnetic shielding performance [1,2]. Among them, Mg-Gd-Y-Zn-Zr rare-earth magnesium alloys exhibit pronounced advantages in strength and thermal stability, which are mainly attributed to the synergistic effects of alloying elements such as Gd, Y, Zn, and Zr. These elements promote the formation of multiple strengthening phases, including long-period stacking ordered (LPSO) structures, rare-earth-rich phases, and Zr-rich phases [3,4]. However, magnesium alloys possess a hexagonal close-packed (HCP) crystal structure, and only a limited number of independent slip systems can be activated at room temperature, resulting in insufficient coordination ability during plastic deformation [5]. In addition, the size, morphology, and distribution of second phases in rare-earth magnesium alloys exert significant effects on crack initiation, stress concentration, and load transfer behavior [6]. When coarse blocky second phases are inhomogeneously distributed along grain boundaries or the extrusion direction, they tend to weaken the continuity of the matrix and induce localized deformation instability, thereby restricting further improvement in the overall mechanical properties of the material [7].

For Mg-Gd-Y-Zn-Zr rare-earth magnesium alloys, the LPSO phase serves not only as an important strengthening phase but also as a key microstructural constituent governing plastic deformation behavior [4]. An appropriate amount of finely dispersed LPSO phase is beneficial for improving the load-bearing capacity of the alloy and enhancing deformation compatibility. In contrast, coarse blocky or continuously distributed LPSO phases may act as preferential sites for stress concentration and crack initiation [8,9]. In addition, the size and distribution of rare-earth-rich phases and Zr-rich phases also affect grain boundary stability and local strain partitioning [10]. Therefore, improving the distribution of second phases, reducing microstructural inhomogeneity, and enhancing deformation coordination through appropriate external-field regulation remain critical issues for optimizing the microstructure and properties of rare-earth magnesium alloys.

Electropulsing treatment, as an emerging external-field regulation technique for materials, has been widely applied in recent years for the microstructural modification and property optimization of metallic materials [11,12]. When pulsed current is applied to metallic materials, coupled effects such as Joule heating and electroplasticity may occur. Joule heating can directly increase the sample temperature and may promote recovery, recrystallization, and the precipitation/dissolution of second phases, whereas electroplasticity, which is commonly associated with non-thermal effects such as electron wind force, may assist dislocation motion and atomic migration [13,14]. Compared with conventional heat treatment, electropulsing treatment is characterized by a rapid heating rate, concentrated energy input, and short processing duration, making it possible to achieve microstructural regulation within a relatively short time [15,16]. For Mg-Gd-Y-Zn-Zr rare-earth magnesium alloys, conventional heat treatment generally relies on relatively long thermal exposure to promote solute diffusion, recovery/recrystallization, and the morphological regulation of LPSO phases and RE-rich second phases. In contrast, electropulsing treatment can introduce rapid thermal input through a short-duration pulsed current, which may accelerate second-phase evolution and local defect rearrangement, thereby providing a potential route for rapid microstructural regulation of such alloy wires.

However, the effectiveness of electropulsing treatment is closely related to processing parameters, such as current density, frequency, duty cycle, and treatment duration [17,18]. Among these parameters, current density directly determines the Joule heating input and electric-field intensity inside the material and further affects dislocation motion, atomic diffusion, grain boundary migration, and phase stability [18]. Previous studies have demonstrated that appropriate pulsed current input can facilitate microstructural recovery, recrystallization, and second-phase regulation in metallic materials; nevertheless, excessive energy input may also induce grain coarsening, abnormal precipitation of second phases, or deterioration of microstructural stability [19]. Therefore, clarifying the microstructural response and mechanical-property evolution of rare-earth magnesium alloys under different current densities is of great significance for determining the effective processing window of electropulsing treatment and avoiding microstructural degradation caused by excessive thermal input. In particular, for Mg-Gd-Y-Zn-Zr rare-earth magnesium alloy wires containing multiple second phases, such as LPSO phases, rare-earth-rich phases, and Zr-rich phases, the correlations among second-phase dissolution, re-precipitation, and mechanical response under different current densities remain to be further elucidated.

Based on this, extruded Mg-10Gd-3.4Y-1.3Zn-0.4Zr rare-earth magnesium alloy wire was selected as the research material in this study. It is hypothesized that a moderate current density is beneficial for LPSO phase fragmentation and second-phase homogenization, whereas an excessively high current density may induce W-phase precipitation, grain coarsening, and mechanical degradation. To verify this hypothesis, electropulsing treatments were conducted at three current densities of 12, 15, and 20 A/mm^2^ under fixed duty cycle and frequency conditions. The temperature-rise behavior, second-phase evolution, microstructural characteristics, and mechanical-property response of the alloy under different current densities were systematically investigated. Particular attention was paid to the evolution of LPSO phases, rare-earth-rich phases, and Zr-rich phases under low, medium, and high thermal-input conditions. The influence mechanism of pulsed current density on microstructural stability and mechanical degradation behavior in rare-earth magnesium alloys was clarified, providing a theoretical basis for electropulsing-assisted microstructural regulation and subsequent optimization of plastic forming processes for rare-earth magnesium alloys.

## 2. Materials and Methods

### 2.1. Materials

An extruded Mg-10Gd-3.4Y-1.3Zn-0.4Zr (wt.%) rare-earth magnesium alloy wire with a diameter of 5 mm was used as the experimental material in this study, and its chemical composition is listed in Table 1. The wire billet was prepared by semi-continuous casting. After casting, the ingot was directly hot-extruded at 480–500 °C, with an extrusion speed of 0.2–0.4 mm/s and an extrusion ratio of 23:1. After extrusion, the wire was air-cooled to room temperature. To avoid the adverse effects of surface impurities and contaminants on the electropulsing experiment, the wire surfaces were ground with 600#–2000# SiC papers prior to treatment.

### 2.2. Experimental Methods

**Figure 1:**
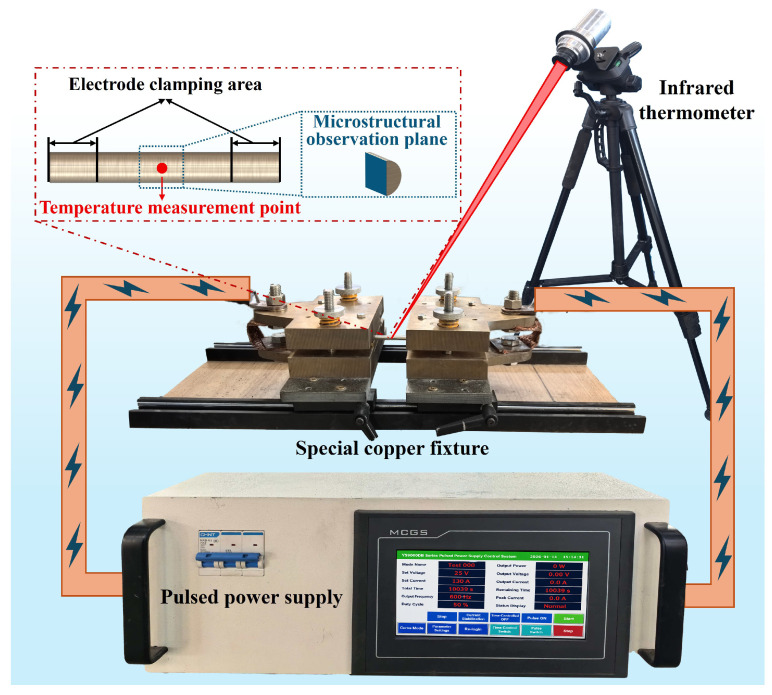
Schematic diagram of the electropulsing experimental setup.

The experimental procedure was as follows: First, wire samples with a length of 50 mm were ground smooth using SiC papers to remove remaining cutting fluid residues and oxide layers from wire electrical discharge machining. Second, the ground wire samples were fixed using a specially designed copper fixture and connected to a YS9000DB.30300 digital programmable pulsed power supply (Shanghai Yisheng Electronic Technology Co., Ltd., Shanghai, China) to form a closed circuit. The pulsed current parameters are listed in Table 2, and the connection configuration is shown in Figure 1. Third, the knurled nuts were tightened to ensure close contact between the wire and the fixture. 

During electropulsing treatment, the wire temperature was monitored using an infrared thermometer (Wuxi Shiao Technology Co., Ltd., Wuxi, China). The thermometer was aimed at the exposed middle region between the two electrode clamping areas, as shown in Figure 1. The measurement position, distance, and angle were kept consistent for all samples. The maximum recorded value was defined as the measured surface peak temperature.

### 2.3. Microstructural Characterization

The samples were sectioned using a DK7740T wire electrical discharge machining (WEDM) device (Taizhou Gangyang Longyu Machinery Factory, Taizhou, China) along a plane parallel to the extrusion direction, and the cut surface was used for microstructural observation. After grinding with SiC papers and mechanical polishing, the samples were etched using a solution consisting of 100 mL absolute ethanol, 10 mL distilled water, 4 mL glacial acetic acid, and 5 g picric acid. The microstructures were then observed using a Leica DM2700M optical microscope (Leica Microsystems, Wetzlar, Germany). Phase identification was performed using an X-ray diffractometer (XRD, D8 Advance, Bruker AXS GmbH, Karlsruhe, Germany) with a scanning range of 10–80° and a scanning rate of 5°/min. The microstructures were further examined using a SIGMA HD scanning electron microscope (SEM; Carl Zeiss Microscopy GmbH, Oberkochen, Germany) equipped with a 51-XMX1003 energy-dispersive X-ray spectrometer (EDS; Oxford Instruments, Abingdon, UK). The accelerating voltage was set to 20 kV, and the spot size was set to 6.0. To analyze grain structure, local strain distribution, and crystallographic orientation of the samples, electron backscatter diffraction (EBSD) was conducted using a Quanta FEG 450 field-emission scanning electron microscope (FE-SEM; FEI Company, Hillsboro, OR, USA). The step size was set to 0.3 μm, and the EBSD data were analyzed using AZtecCrystal software (version 2.1, Oxford Instruments, Abingdon, UK).

### 2.4. Mechanical Property Testing

Tensile tests were conducted at room temperature using an Instron 5969 universal testing machine (Instron, Norwood, MA, USA). The tensile specimens were machined along the wire axial direction according to GB/T 228.1-2021 [20]. Each tensile specimen had a total length of 50 mm, a gauge length of 25 mm, and a gauge diameter of 4.5 mm. To minimize the influence of surface defects on the test results, the specimen surfaces were progressively ground with 600#–2000# SiC papers before testing until a smooth surface was obtained. This procedure was adopted to avoid additional stress concentration caused by burrs or scratches during loading and thus improve the reliability of the test data. The loading rate was set to 0.5 mm/min.

## 3. Results

### 3.1. Mechanical Properties and Temperature-Rise Characteristics

**Figure 2:**
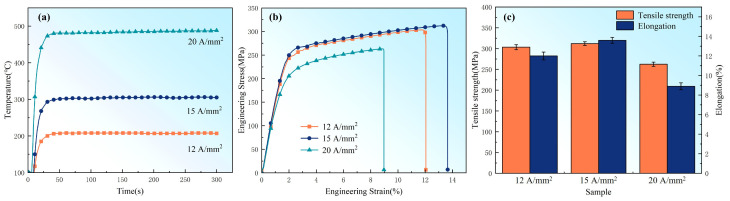
(**a**) Temperature-rise curves; (**b**) room-temperature tensile stress–strain curves; (**c**) corresponding tensile properties.

Table 3 presents the mechanical properties and peak temperatures of the alloy after treatment at different pulsed current densities. Figure 2 shows the temperature-rise curves during electropulsing treatment, the room-temperature tensile stress–strain curves, and the corresponding tensile properties of the alloy under different current densities. It can be observed that the peak temperature of the samples increased significantly as the current density increased from 12 to 20 A/mm^2^, indicating that current density has a direct influence on the Joule heating input during electropulsing treatment.

At a current density of 12 A/mm^2^, the peak temperature of the sample was 207 °C, and the ultimate tensile strength and elongation were 303.5 MPa and 12.0%, respectively. Under this condition, the thermal input was relatively low, resulting in a limited regulatory effect on the alloy microstructure; therefore, the variation in mechanical properties was relatively small. When the current density increased to 15 A/mm^2^ the peak temperature rose to 302 °C, while the ultimate tensile strength and elongation increased to 312.2 MPa and 13.6%, respectively. This indicates that a moderate pulsed current input is beneficial for improving deformation coordination, thereby enabling the alloy to achieve a relatively favorable strength–ductility combination. However, when the current density was further increased to 20 A/mm^2^, the peak temperature sharply increased to 497 °C, while the ultimate tensile strength and elongation decreased to 262.5 MPa and 8.9%, respectively. This suggests that excessive thermal input induced by an excessively high current density reduces microstructural stability, thereby weakening both the load-bearing capacity and plastic deformation ability of the material.

Overall, the mechanical properties and temperature-rise curves demonstrate that the performance differences of the alloy under different pulsed current densities are closely associated with the thermal input level. At 12 A/mm^2^, the microstructural response was insufficient. At 15 A/mm^2^, the thermal input was relatively moderate, which was favorable for second-phase regulation and deformation coordination. In contrast, the excessive temperature rise at 20 A/mm^2^ may induce grain coarsening and second-phase re-precipitation, ultimately resulting in the simultaneous deterioration of strength and ductility.

### 3.2. Microstructural Characteristics

**Figure 3:**
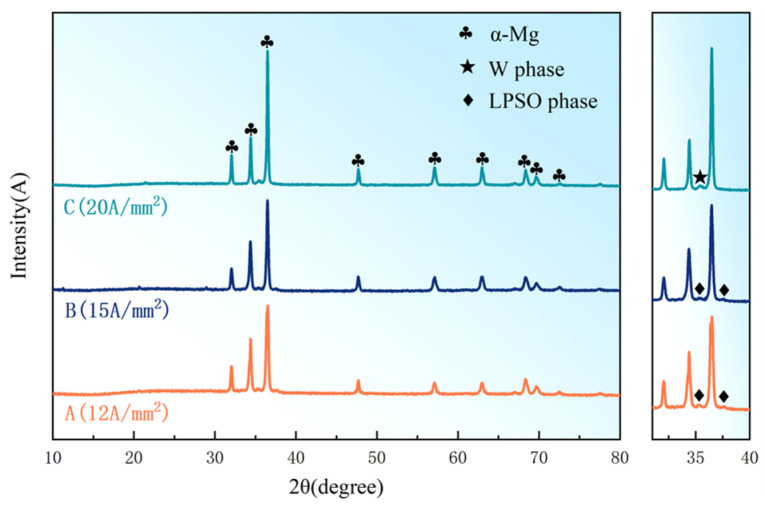
XRD patterns of the alloy treated at different current densities.

Figure 3 shows the XRD patterns of the Mg-10Gd-3.4Y-1.3Zn-0.4Zr alloy samples after electropulsing treatment at different current densities. The XRD patterns show that the alloy samples treated at 12 A/mm^2^ and 15 A/mm^2^ are mainly composed of α-Mg and LPSO phases. The intensity of the LPSO-related diffraction peaks is weakened in the 15 A/mm^2^ sample. Due to the relatively low fraction of fine RE-rich phases, These phases were not detected by XRD. In contrast, the alloy sample treated at 20 A/mm^2^ was mainly composed of α-Mg and W phases. These results suggest that the phase constitution and relative phase content varied after electropulsing treatment under different processing parameters.

**Figure 4:**
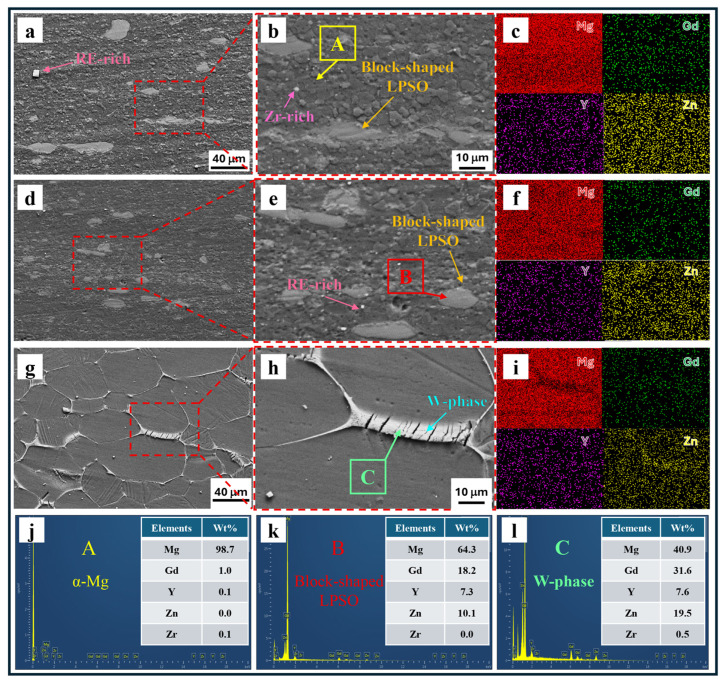
SEM-BSE/EDS images of the alloy treated at different current densities: (**a**–**c**) 12 A/mm^2^; (**d**–**f**) 15 A/mm^2^; (**g**–**i**) 20 A/mm^2^; (**j**–**l**) corresponding EDS results of selected regions A–C (wt.%).

Figure 4 shows the SEM-BSE/EDS images of the alloy after electropulsing treatment at different pulsed current densities. In the sample treated at 12 A/mm^2^, a relatively large number of blocky LPSO phases can be observed, and the second phases are relatively coarse and inhomogeneously distributed. The EDS result in Figure 4j shows that region A contains 98.7 wt.% Mg, indicating that this region corresponds to the α-Mg matrix. When the current density was increased to 15 A/mm^2^, the blocky LPSO phases underwent partial dissolution and fragmentation. Meanwhile, the observed Zr-rich particles changed from a coarse and unevenly distributed population to relatively fine and dispersed particles. These results indicate that a moderate current density can promote the fragmentation, partial dissolution, and more homogeneous dispersion of second phases, which is beneficial for improving microstructural homogeneity. The EDS result in Figure 4k shows that region B is enriched in Gd, Y, and Zn, confirming that this region corresponds to the blocky LPSO phase.

When the current density was increased to 20 A/mm^2^, the alloy microstructure changed markedly. A bright white second phase with a network-like distribution appeared along the grain boundaries, and large rare-earth-rich phases were also observed to re-precipitate. The EDS result in Figure 4i shows that region C exhibits significantly increased contents of Gd, Y, and Zn. Combined with the XRD results, this phase can be identified as the W phase. Compared with the samples treated at 12 and 15 A/mm^2^, the second phases under the 20 A/mm^2^ condition became coarser and more discontinuously distributed, indicating that excessive Joule heating input disrupted the previous trend of second-phase dissolution and dispersion, causing the microstructure to evolve toward a coarsened and inhomogeneous state. Such coarse second phases and their continuous distribution along grain boundaries can readily induce local stress concentration, which is an important reason for the deterioration of mechanical properties [8].

**Figure 5:**
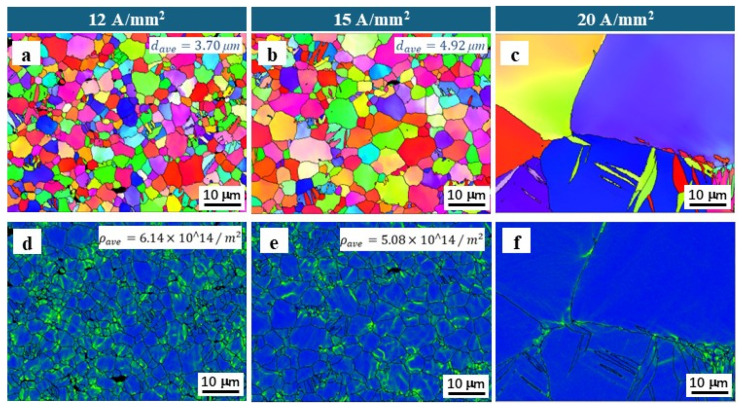
EBSD maps of the alloy treated at different current densities: (**a**–**c**) IPF maps; (**d**–**f**) GND maps.

Figure 5 shows the EBSD-IPF maps and GND distribution maps of the alloy after electropulsing treatment at different pulsed current densities. It can be observed that the sample treated at 12 A/mm^2^ is mainly composed of equiaxed grains, with partial grain growth occurring. This indicates that the pulsed current at a low current density already exerted a certain influence on grain boundary migration, although the degree of microstructural homogenization remained limited. In the sample treated at 15 A/mm^2^, although grain growth also occurred, the microstructure became more uniformly distributed, suggesting that moderate thermal input promoted grain adjustment and local microstructural homogenization. Combined with the GND distribution, the dislocation density in the 15 A/mm^2^ sample was reduced, indicating that dislocation recovery and rearrangement were more sufficient and that local strain concentration inside the material was partially alleviated.

In the sample treated at 20 A/mm^2^, grain coarsening became more pronounced, accompanied by a decrease in microstructural homogeneity in local regions. The GND distribution indicates that the local strain state was not further optimized under this condition, suggesting that excessive thermal input reduced the microstructural stability. Combined with the SEM results, the re-precipitation of coarse second phases and grain coarsening under the 20 A/mm^2^ condition jointly weakened the deformation compatibility of the material, resulting in the simultaneous deterioration of strength and ductility. Owing to the significant grain coarsening in the 20 A/mm^2^ sample, the number of effective grains within a single EBSD field of view was relatively limited, leading to insufficient statistical representativeness. Therefore, the EBSD results under this condition were mainly used to reflect the characteristics of local grain coarsening and microstructural inhomogeneity, rather than for rigorous quantitative comparison of crystallographic orientation or GND density.

**Figure 6:**
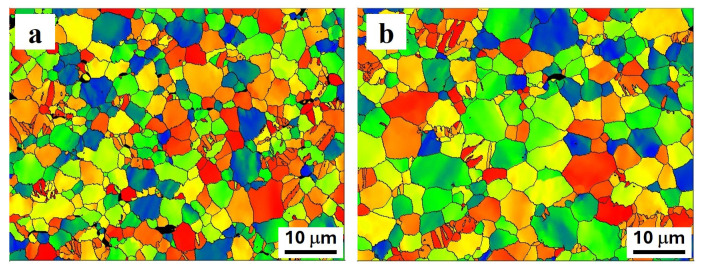
Schmid factor distribution maps for <c+a> non-basal slip in the alloy treated at different current densities: (**a**) 12 A/mm^2^; (**b**) 15 A/mm^2^.

To further reveal the effect of grain orientation on slip activation in the samples after electropulsing treatment at different current densities, the Schmid factor (SF) distributions along the loading direction were analyzed, as shown in Figure 6. Slip is the dominant plastic deformation mechanism in magnesium alloys and mainly depends on <a> slip at room temperature, which primarily corresponds to basal slip of <11$\bar{2}$0>{0001}. However, basal slip provides only two independent slip systems, resulting in the poor room-temperature deformability of magnesium alloys [21,22]. The addition of rare-earth elements such as Gd and Y can activate <c+a> slip in magnesium alloys, mainly corresponding to <11$\bar{2}$3>{11$\bar{2}$2} slip. The activation of <c+a> slip is an important factor contributing to enhanced softening behavior and reduced flow stress [23]. A higher proportion of grains with SF > 0.4 indicates that the corresponding slip systems are more readily activated [24].

The variation in the Schmid factor distribution for basal slip with SF > 0.4 was relatively small with increasing current density, indicating that pulsed current density had a limited effect on the crystallographic orientation favorability for basal slip. In contrast, the Schmid factor distribution for <c+a> non-basal slip with SF > 0.4 was more sensitive to current density. Among the samples, the 15 A/mm^2^ sample exhibited a higher proportion of grains with SF > 0.4, suggesting that more grains under this condition possessed favorable orientations for non-basal slip activation along the loading direction. The activation of non-basal slip contributes to improving the plastic deformation compatibility of HCP magnesium alloys, which is one of the important reasons for the increased elongation of the 15 A/mm^2^ sample. Owing to the significant grain coarsening in the 20 A/mm^2^ sample, the number of effective grains within a single EBSD field of view was relatively limited, resulting in insufficient statistical representativeness. Therefore, this study mainly compares and analyzes the Schmid factor distributions of the 12 and 15 A/mm^2^ samples.

## 4. Discussion

During pulsed current treatment, the current density directly affects the Joule heating input inside the sample, further altering the stability of second phases, grain structure, and local deformation compatibility of the alloy. As indicated by the temperature-rise curves, the peak temperature increased from 207 °C to 497 °C as the current density increased from 12 to 20 A/mm^2^, demonstrating that increasing current density significantly intensified the Joule heating effect during electropulsing treatment. Under different thermal-input conditions, the alloy exhibited distinct microstructural evolution pathways, accompanied by pronounced differences in mechanical properties. At 12 A/mm^2^, the peak temperature was relatively low, and the regulatory effect of pulsed current on second phases and grain structure was limited. The blocky LPSO phases remained relatively coarse and inhomogeneously distributed. Therefore, although the sample retained a certain degree of ductility under this condition, coarse second phases and microstructural inhomogeneity still induced local stress concentration, resulting in only limited improvement in the strength–ductility combination.

When the current density was increased to 15 A/mm^2^, the peak temperature of the sample rose to 302 °C, indicating that the thermal input reached a relatively moderate level. Under this condition, the blocky LPSO phases underwent dissolution and fragmentation, while the Zr-rich phases gradually transformed from large particles into fine and dispersed particles. The reduced size and improved distribution uniformity of the second phases helped decrease stress concentration at grain boundaries and phase interfaces, thereby making load transfer and plastic deformation more homogeneous. This second-phase distribution state could not only maintain the strengthening effect to a certain extent but also weaken the adverse influence of coarse second phases on plastic deformation. As a result, the 15 A/mm^2^ sample exhibited relatively superior mechanical properties among the three conditions, with an ultimate tensile strength and elongation of 312.2 MPa and 13.6%, respectively.

The difference in second-phase evolution between the 15 A/mm^2^ and 20 A/mm^2^ conditions is mainly related to the thermal input level and the thermal stability of the LPSO and W phases. At 15 A/mm^2^, the moderate thermal input promoted local diffusion of Gd, Y, and Zn solute atoms and recovery, leading to the partial dissolution, fragmentation, and redistribution of blocky LPSO phases. However, this thermal input was still insufficient to induce severe grain coarsening or massive re-precipitation of coarse second phases; therefore, the overall microstructure tended to become more homogeneous. In contrast, when the current density increased to 20 A/mm^2^, the measured surface peak temperature reached 497 °C. The excessive thermal input significantly enhanced the diffusion of Gd, Y, and Zn atoms and changed the local solute distribution near grain boundaries, reducing the stability of the original LPSO phase and making the precipitation of W phase or RE-rich phases more favorable. Grain boundaries have relatively high interfacial energy and also act as fast diffusion paths and preferential nucleation sites; therefore, coarse W phase or RE-rich phases tended to precipitate along grain boundaries and form a network-like distribution. Meanwhile, the high thermal input also enhanced grain-boundary migration, resulting in grain coarsening.

**Figure 7:**
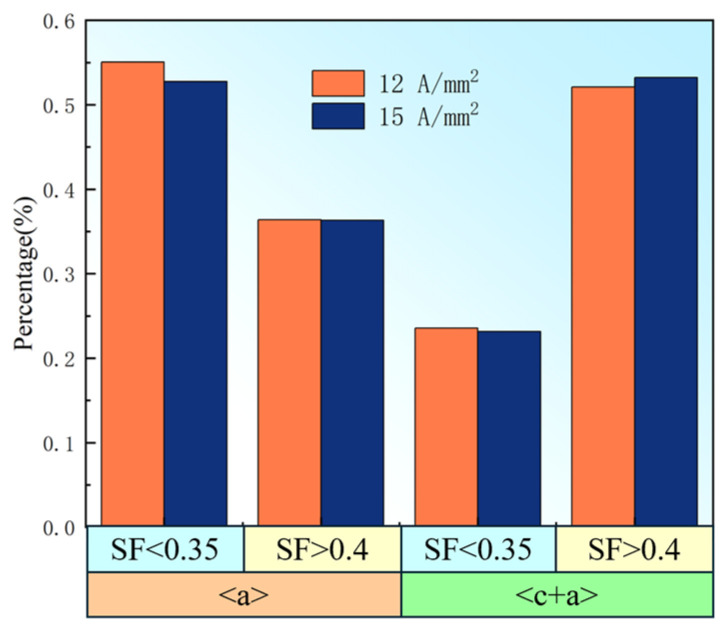
Schmid factor maps of the alloy after electropulsing treatment.

In addition to second-phase evolution, moderate thermal input also affects the dislocation state and grain-orientation characteristics of the alloy. The EBSD results show that the high-GND-density regions decreased in the 15 A/mm^2^ sample, indicating that dislocation recovery and rearrangement were more sufficient under this condition and that local strain concentration was partially alleviated. For HCP magnesium alloys, basal slip is readily activated at room temperature; however, the limited number of independent slip systems provided by basal slip alone makes it difficult to fully accommodate complex plastic deformation. Therefore, the participation of non-basal slip plays an important role in improving ductility. To further reveal the effect of grain orientation on slip activation after electropulsing treatment at different current densities, the Schmid factor (SF) distributions along the loading direction were analyzed, as shown in Figure 7. The proportion of grains with high Schmid factors for <c+a> slip was significantly increased in the 15 A/mm^2^ sample (reaching 53.2%), indicating that more grains acquired favorable orientations for non-basal slip activation. It should be noted that the Schmid factor reflects the geometrical favorability of slip activation rather than the critical resolved shear stress itself. Therefore, the improved elongation under the 15 A/mm^2^ condition can be attributed to the synergistic effects of second-phase homogenization, dislocation recovery, and enhanced orientation favorability for non-basal slip.

It should be noted that the increase in tensile strength of the 15 A/mm^2^ sample did not originate from grain-size strengthening. Compared with the 12 A/mm^2^ sample, the 15 A/mm^2^ sample exhibited a certain degree of grain growth, which may weaken the grain-boundary strengthening effect. However, the microstructural improvement induced by moderate thermal input played a dominant role in enhancing the mechanical properties. The partial dissolution and fragmentation of blocky LPSO phases, together with the more homogeneous distribution of second phases, helped reduce local stress concentration and crack-initiation sensitivity. Meanwhile, the improved matrix continuity and reduced local strain incompatibility were beneficial for load transfer and deformation coordination. Therefore, although moderate grain growth occurred, the strengthening and damage-suppression effects induced by second-phase refinement and improved deformation compatibility dominated the mechanical response, enabling the 15 A/mm^2^ sample to achieve higher tensile strength and elongation.

When the current density was further increased to 20 A/mm^2^, the peak temperature of the sample sharply rose to 497 °C. The excessive Joule heating input caused the microstructural evolution to deviate from the optimized pathway of second-phase dissolution and dispersion. The SEM-BSE/EDS results show that a bright network-like W phase appeared along the grain boundaries in the 20 A/mm^2^ sample, accompanied by the re-precipitation of large rare-earth-rich phases. Unlike the refined and dispersed second phases observed under the 15 A/mm^2^ condition, the grain-boundary second phases in the 20 A/mm^2^ sample were coarser and exhibited a network-like distribution. These features could readily weaken the continuity of the α-Mg matrix and become preferential sites for interfacial debonding, microcrack initiation, and crack propagation under external loading. In addition, the high thermal input also led to pronounced grain coarsening, reducing the grain boundary area and weakening intergranular deformation compatibility. It should be noted that, due to the obvious grain coarsening in the 20 A/mm^2^ sample, the number of effective grains within a single EBSD field of view was limited. Therefore, the GND density and Schmid factor results of this sample were not used for rigorous quantitative comparison, but only as qualitative evidence to illustrate grain coarsening and microstructural inhomogeneity under high thermal input. The conclusion that deformation compatibility decreased at high current density is mainly supported by the combined results of SEM-BSE/EDS, XRD, temperature-rise curves, and tensile properties. The precipitation of coarse grain-boundary W phase/RE-rich phases, grain coarsening, and the simultaneous decrease in strength and elongation collectively indicate the deterioration of microstructural stability and deformation compatibility under the 20 A/mm^2^ condition.

**Figure 8:**
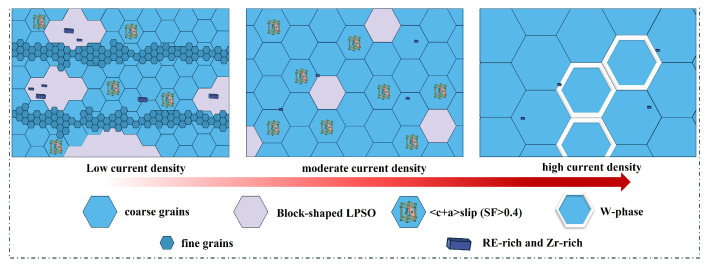
Schematic illustration of the microstructural evolution mechanism induced by electropulsing treatment under different thermal input conditions.

Overall, the effect of pulsed current density on Mg-Gd-Y-Zn-Zr rare-earth magnesium alloy wires is not a monotonic strengthening process, but instead shows an appropriate thermal-input process window under the conditions investigated in this study. As shown in Figure 8, at low current density, the thermal input is insufficient, resulting in limited second-phase dissolution and microstructural homogenization. At moderate current density, the partial dissolution/fragmentation of blocky LPSO phases, homogenized distribution of second phases, dislocation recovery, and improved orientation favorability for potential <c+a> non-basal slip jointly improve the deformation compatibility of the material, enabling the alloy to achieve a relatively good strength–ductility balance. In contrast, at excessively high current density, excessive thermal input induces grain coarsening and the re-precipitation of W phase or RE-rich second phases, disrupting the previously formed microstructural homogenization trend and ultimately reducing the tensile strength and elongation to 262.5 MPa and 8.9%, respectively. Therefore, in electropulsing treatment of rare-earth magnesium alloy wires, reasonable control of current density and thermal input level is the key to achieving second-phase regulation, microstructural stabilization, and improved mechanical properties.

## 5. Conclusions

In this study, Mg-10Gd-3.4Y-1.3Zn-0.4Zr rare-earth magnesium alloy wires were used as the research material. The temperature-rise behavior, second-phase evolution, microstructural characteristics, and mechanical response of the alloy under different pulsed current densities were investigated. The main conclusions are as follows:

(1) Pulsed current density significantly affected the thermal input level and mechanical properties of the alloy. As the current density increased from 12 A/mm^2^ to 20 A/mm^2^, the measured surface peak temperature increased from 207 °C to 497 °C. Among the tested conditions, the 15 A/mm^2^ sample exhibited relatively better mechanical properties, with tensile strength and elongation of 312.2 MPa and 13.6%, respectively. In contrast, due to excessive thermal input, the tensile strength and elongation of the 20 A/mm^2^ sample decreased to 262.5 MPa and 8.9%, respectively.
(2) A moderate current density of 15 A/mm^2^ was beneficial for second-phase regulation and microstructural homogenization. The blocky LPSO phases underwent dissolution and fragmentation, while the Zr-rich phases became finer and tended to be more dispersed. Meanwhile, the dislocation density decreased. Schmid factor analysis indicated that the orientation distribution under this condition was more favorable for potential <c+a> non-basal slip activation. In contrast, an excessively high current density of 20 A/mm^2^ reduced microstructural stability. Obvious grain coarsening occurred, accompanied by the re-precipitation of W phase and RE-rich phases along grain boundaries, which weakened matrix continuity and induced stress concentration.
(3) The effect of pulsed current density on Mg-Gd-Y-Zn-Zr rare-earth magnesium alloy wires was not a monotonic strengthening process, but showed an obvious thermal-input-dependent process window. A moderate current density promoted second-phase dissolution/dispersion, dislocation recovery, and improved deformation compatibility. However, an excessively high current density caused microstructural coarsening and re-precipitation of W phase or RE-rich second phases, ultimately leading to simultaneous decreases in strength and ductility. Therefore, electropulsing treatment of rare-earth magnesium alloy wires should be controlled within an appropriate current-density and thermal-input window to achieve second-phase homogenization while avoiding microstructural coarsening and phase instability.

## Figures and Tables

**Table 1:** Extruded Mg-Gd-Y-Zn-Zr alloying element composition (wt.%).

Composition	Gd	Y	Zn	Zr	Mg
**Content**	10.06	3.35	1.25	0.36	Bal.

**Table 2:** Experimental parameters of the pulsed power supply.

Sample No.	Current Density (A/mm^2^)	Current Frequency (Hz)	Duty Cycle (%)	Treatment Time (s)	Pulse Width (µs)
A	12	600	40	300	667
B	15	600	40	300	667
C	20	600	40	300	667

**Table 3:** Mechanical properties and peak temperatures of the alloy treated at different current densities.

Sample No.	Current Density (A/mm^2^)	Ultimate Tensile Strength (MPa)	Yield Strength (MPa)	Elongation (%)	Uniform Elongation (%)	Peak Temperature (°C)
A	12	303.5	229.5	12.0	11.7	207
B	15	312.2	239.6	13.6	13.3	302
C	20	262.5	167.4	8.9	8.8	497

## Data Availability

The original contributions presented in this study are included in the article. Further inquiries can be directed to the corresponding author.
